# The effects of hip strengthening exercises in a patient with plantar fasciitis: A case report

**DOI:** 10.1097/MD.0000000000016258

**Published:** 2019-06-28

**Authors:** Jin Hyuck Lee, Jong Hoon Park, Woo Young Jang

**Affiliations:** a Department of Sports Medical Center, Korea University College of Medicine, Anam Hospital; b Department of Orthopedic Surgery, College of Medicine, Korea University, Seoul, Republic of Korea.

**Keywords:** abductor muscles, joint mobilization, plantar fasciitis, plantarflexion muscles, strengthening exercise

## Abstract

**Rationale:**

Plantar fasciitis is a common cause of foot pain presenting with morning stiffness and plantar area pain. This case study is to optimize the management in patient with plantar fasciitis accompanied by apparent high-arch foot.

**Patient concerns:**

A 55-year-old women presented with plantar fasciitis accompanied by apparent high-arch foot. The pain presents for the heel and pelvic areas with long-distance walking.

**Diagnoses:**

She was diagnosed with plantar fasciitis accompanied by apparent high-arch foot for physical examination and plain radiographs. In muscle performance and dynamic postural stability tests, indicated the muscle weakness and postural instability.

**Interventions:**

The patient was treated with manual therapy such as joint and soft tissue mobilization interventions including plantar fascia and gastrocnemius stretching, but the heel and pelvic pain were aggravated during long-distance walking. After hip strengthening exercises, the heel and pelvic pain significantly improved.

**Outcomes:**

The 3-month follow-up revealed that the heel and pelvic pain did not occur in the long-distance walking, and there was no pain and discomfort at one year follow-up.

**Lessons:**

To optimize the management in patient with plantar fasciitis accompanied by apparent high-arch deformity, clinicians should try to identify the hip abductor muscles weakness, and therapists should consider incorporating hip strengthening exercises.

## 1 Introduction

Plantar fasciitis (PF), one of the most common causes of foot pain and presents with morning stiffness and plantar area foot pain.^[1]^ Previous studies have reported various risk factors for PF, including limited dorsiflexion of the ankle joint, excessive use, high body mass index, poor shock absorption, and foot posture.^[2,3]^ Among them, limited dorsiflexion of the ankle joint is caused by tightness of the gastrocnemius muscle^[4,5]^ and is reported to be a primary cause of PF.^[2]^ To date, physical therapy clinical practice guidelines have emphasized the importance of manual therapies such as joint mobilization and soft tissue mobilization to improve limited ankle range of motion (ROM).^[2]^ However, recent studies have reported that PF was not directly related to limited ankle ROM.^[6,7]^

Since PF is associated with foot posture,^[1,3,8,9]^ strengthening exercises are often recommended to address excessive foot pronation and supination, which can increase the stress on the soft tissue structures and plantar fascia. Recent studies have reported that foot postures such as apparent low-arch foot (pronated) and high-arch foot (supinated) were associated with weakness of the hip abductor muscles,^[10,11]^ since the hip muscles play an important role in lower limb biomechanics. Herein, we report a case of PF with apparent high-arch foot that was successfully treated by hip strengthening exercises. The patient was informed that data concerning the case would be submitted for publication of the case, and she provided consent.

## 2 Case presentation

A 55-year-old women (height: 156 cm, weight: 62 kg) presented with right foot pain that developed 1 year ago. The initial symptoms began in the forefoot and heel regions, and she had received a one-time steroid injection at another hospital. Thereafter, there was a slight decrease in forefoot pain. However, the pain rapidly increased 5 months after the steroid injection and was focused in the heel area. The heel pain was aggravated immediately after rest, such as while getting out of bed in the morning. She also complained of intermittent right pelvic pain without radiculopathy and low back pain. The pelvic pain was aggravated with prolonged standing or after long-distance walking and decreased with rest. Although there was only minor heel pain during weight-bearing, it was aggravated with single-leg weight-bearing. The heel area and the right-side pelvic pain showed a visual analogue scale (VAS) of 8 and 3 during single-leg weight-bearing, respectively.

**Figure 1:**
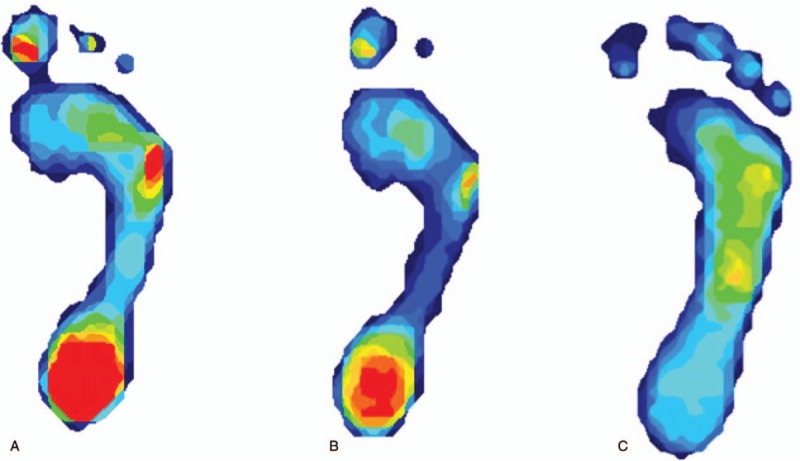
Pedobarograph testing: (A) Pre-intervention and (B) gastrocnemius stretching exercises only showed foot pressure concentrated on the lateral forefoot and heel area. (C) Foot pressure after hip strengthening exercises with manual therapy showed a remarkable change in the lateral forefoot and heel area.

**Figure 2:**
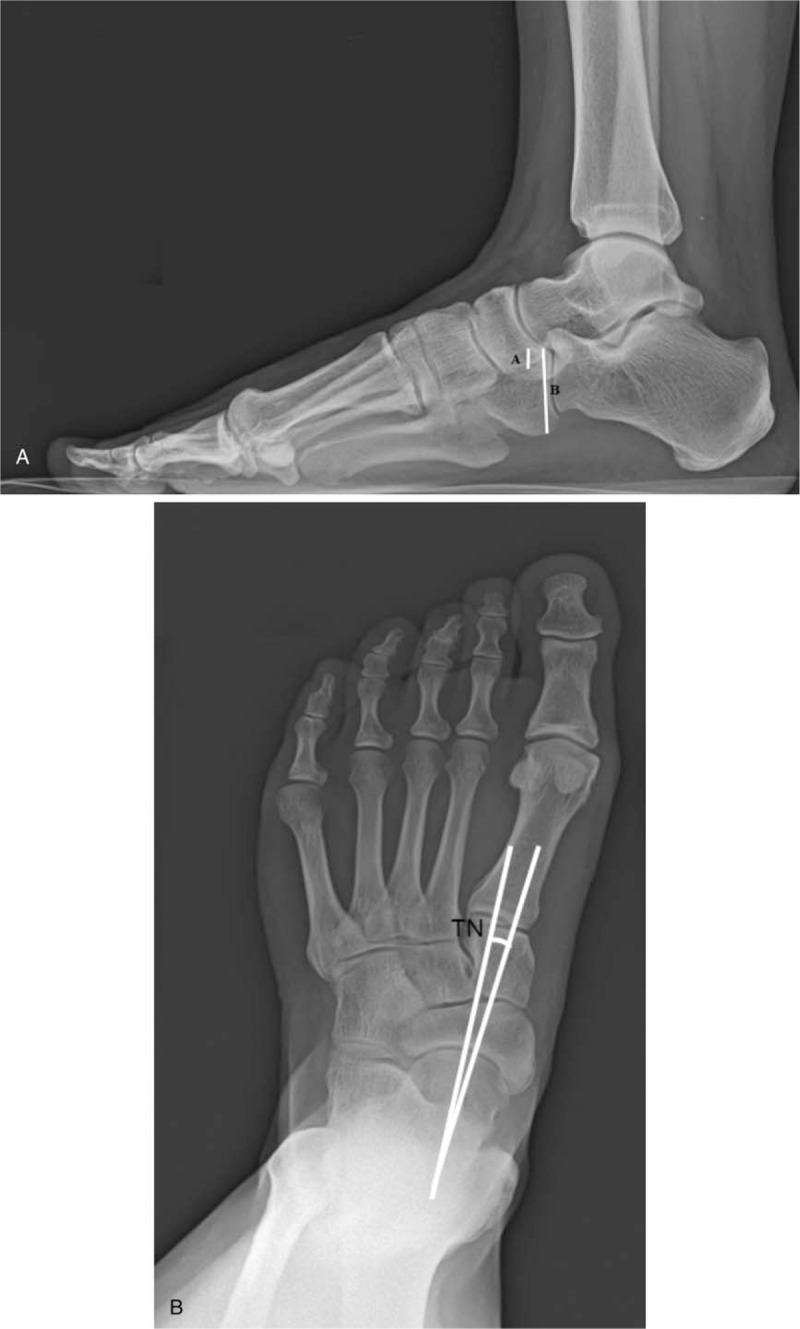
(A) Naviculocuboid overlap and (B) talonavicular coverage angle on plain radiographs showed a normal-arch foot.

Physical examination showed tenderness centered on the fifth metatarsal head and the medial tubercle of the calcaneus. The navicular height test^[12]^ and a pedobarograph system^[13]^ were used to measure the foot arch and foot pressure (Fig. 1A), respectively. Gastrocnemius contracture was measured by the Silfverskiold test^[14]^ and defined as limited dorsiflexion ROM with knee extension and normal dorsiflexion ROM with knee flexion (Table 1). Plain radiographs revealed a naviculocuboid overlap and talonavicular coverage angle of 26.9% and 1.7°, retrospectively, which were representative of a normal arch foot (Fig. 2).^[15,16]^ However, since this patient had a high-arch foot posture, we defined it as apparent high-arch foot without cavovarus deformity.

**Table 1:**
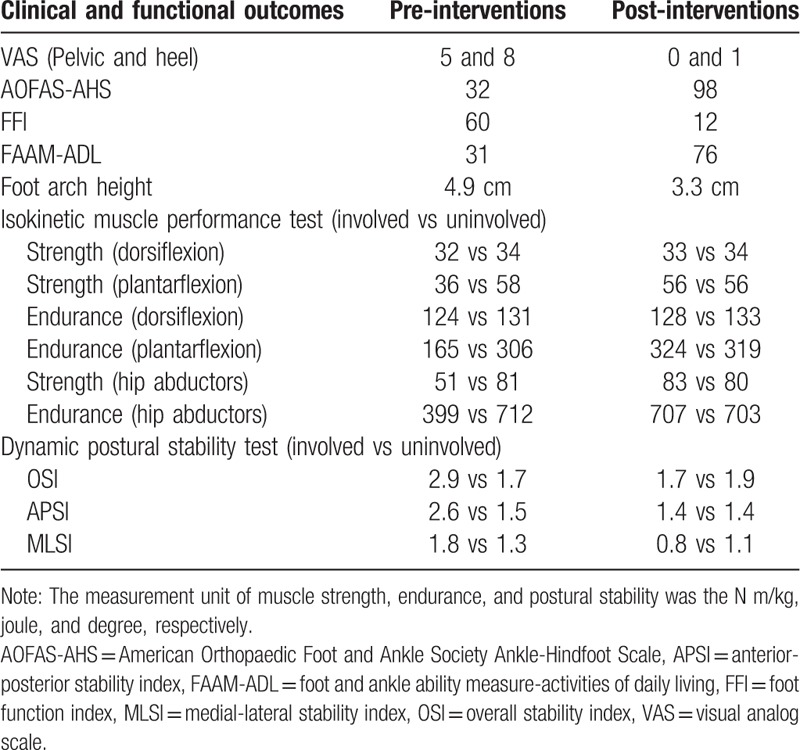
Clinical and functional outcomes.

Foot function was measured using foot questionnaires such as the American Orthopedic Foot and Ankle Society Ankle-Hindfoot Scale (AOFAS-AHS), Foot Function Index (FFI), and activities of daily living (ADL) subscale of the Foot and Ankle Ability Measure (FAAM).^[17,18]^ AOFAS-AHS and FFI were divided into 3 subcategories of pain (40 points), function (50 points), and alignment (10 points), and pain (50 points), disability (90 points), and activity limitation (30 points), respectively. FAAM ADL consisted of 21 questions (4 points: no difficulty at all, 0 points: unable to do). A lower score in the AOFAS-AHS and FAAM ADL is associated with poor functional activities, while it is the opposite in the FFI. The initial scores were 32 of 100 points in the AOFAS-AHS, 60 of 170 points in the FFI, and 31 of 84 points in the FAAM ADL.

Muscle performance testing was conducted using a quantified isokinetic device (Biodex Multi-Joint System 4, Biodex Medical Systems Inc., Shirley, NY) and performed in a semi-sitting position with a slight knee flexion. The test consisted of muscle strength (5 at 30°/s) and endurance (15 at 120°/s) for dorsiflexion and plantarflexion and hip abductors, with a rest time of 30 s. The dynamic postural stability test was evaluated using the Biodex Stability System (BSS; Biodex Medical Systems). BSS provides a 20° tilt and a 360° rotation of the platform, and the patient was instructed to maintain a single-leg posture while the stability level automatically declined from level 12 (most stable) to level 1 (most unstable). Each test consisted of 2 trials of 20 s each. The muscle performance and dynamic postural stability tests (Table 1) revealed weakness of the plantarflexion muscles and poor dynamic postural stability.

Foot questionnaires were completed and evaluation with the navicular height test, pedobarograph, and Silfverskiold test was performed immediately on the day of the first visit, and the muscle performance and postural stability tests were performed 2 weeks later owing to device failure and repair. All tests were performed before and after the interventions.

### 2.1 Interventions

Initial manual therapy such as joint and soft tissue mobilization interventions^[19]^ (including subtalar traction, subtalar lateral glide, talocrural glide, distal tibiofibular glide, midtarsal glide, plantar fascia mobilization, and gastrocnemius stretching) were performed immediately after the evaluation of the Silfverskiold test. After 20 minutes of manual therapy intervention, heel pain was diminished (VAS 8 to 5). Accordingly, instructions for a self-mobilization intervention to improve dorsiflexion ROM were given to the patient to perform at home. The patient was treated 2 times per week for 2 weeks for 30 minutes, and she performed the home exercise program 3 times per day for 30 minutes. However, heel pain did not improve any further, and pedobarograph examination still showed foot pressure concentrated on the lateral forefoot and heel area (Fig. 1B).^[20]^ Furthermore, the patient complained of gradually increasing right pelvic pain (VAS 3 to 5).

The patient experienced increased calf muscles spasms during muscle performance and dynamic postural stability tests. Therefore, the physiotherapist decided to change the treatment protocol to include strengthening exercises, such as plantarflexion muscle and hip and core muscles training, including the gluteal muscles, along with single-leg postural control reeducation. After the application of strengthening exercises, the symptoms of heel and pelvic pain were significantly diminished during walking and single-leg standing (pelvic: VAS 5 to 2; heel: VAS 5 to 2) and foot pressure on pedobarograph reexamination also showed a remarkable change (Fig. 1C). In addition, when she was visited after 3 months with end of her interventions, the heel and pelvic pain on the right foot did not occur in the long-distance walking (Table 1).

### 2.2 Outcomes

The patient was treated 2 times per a week during the 10 weeks with manual therapy and strengthening exercises. All parameters, including heel and pelvic pain (VAS 8 to 1 and 5 to 0, respectively), AOFAS-AHS, FFI, and FAAM ADL (32 to 98 of 100, 60 to 12 of 170, and 31 to 76 of 84 points, respectively), dorsiflexion ROM (pre: 0°; post: 6°), foot pressure (Fig. 1 A–C), foot arch height (pre: 4.9 cm; post: 3.3 cm), muscle performance (plantarflexion: 36 to 56 at 30°/s, 165 to 324 at 120°/s, hip abductors: 51 to 83 at 30°/s, 399 to 707 at 120°/s), dynamic postural stability [overall stability index (OSI): 2.9 to 1.7; anterior-posterior stability index (APSI): 2.6 to 1.4; medial-lateral stability index (MLSI): 1.8 to 0.8], were remarkably improved compared with those at the first visit (Table 1). Although the heel and pelvic pain on the right foot still occurred after long-distance walking for over 3 hours, the pain was significantly lower than before the interventions. The calf muscle spasms also did not occur during ADL. In addition, when she was visited after 3 months with end of her interventions, the heel and pelvic pain on the right foot did not occurr in the long-distance walking. At 1 year follow-up, there was no pain and discomfort.

## 3 Discussion

The patient in this case study had a previous history of PF treatment and presented with apparent high-arch foot with concurrent intermittent pelvic pain. The initial treatment strategy was manual therapy based on the physical therapy clinical practice guidelines for PF. Although the heel pain was slightly decreased after the initial manual therapy intervention, it still increased during walking and single-leg standing. We believe that the increasing heel pain during walking may have been caused by weakness of the plantarflexion muscles. The main roles of the plantarflexion muscles are to prevent excessive loading on the plantar fascia and to maintain the longitudinal arch.^[7,21,22]^ Therefore, weakness of the plantarflexion muscles can increase the load on the plantar fascia.^[12,23]^ In this patient, the muscle performance test showed plantarflexion muscle weakness, and increased calf muscle spasms were observed during walking, which indicated that plantarflexion muscle weakness was one cause of the aggravated heel pain.

The right pelvic pain in this patient had persistently increased during long-distance walking. We believe this may have been due to weakness of the hip abductor muscles. Previous studies reported that the hip abductor muscles play a significant role in the biomechanics of walking and ankle stability,^[24,25]^ including the function of decreasing forefoot plantar peak pressure during gait.^[25]^ In this patient, increased compensatory mechanisms of the hip joint to decrease forefoot plantar peak pressure from forefoot pain may have led to fatigue and weakness of the gluteal muscles, which resulted in aggravation of the right pelvic pain during walking. Conversely, increased compensatory mechanisms of the ankle joint caused by weakness of the hip abductor muscles may have caused the intractable heel pain.^[25]^ Weakness of the hip abductor muscles during walking may result in plantarflexion muscle overuse,^[24]^ which can increase the load on the plantar fascia.^[22]^ Hip abductor muscles contribute to postural stability in the frontal and sagittal planes of the lower extremities,^[26]^ and weakness can cause Trendelenburg gait, resulting in the lateral shift of concentrated pressure to the forefoot and heel areas. Our patient showed poor dynamic postural stability and Trendelenburg sign, suggesting that the heel and pelvic pain were likely caused by hip abductor muscle weakness. Based on the results of this study, we believe that strengthening exercises, especially for the hip joint, may be an effective method to improve various PF symptoms in patients with pelvic pain.

## 4 Conclusion

This case highlights the importance of hip muscle strengthening for the optimal management of PF patients with apparent high-arch foot and concurrent intermittent pelvic pain. To optimize the management in PF patient with apparent high-arch deformity, clinicians should try to identify the hip abductor muscles weakness, and therapists should consider incorporating hip strengthening exercises.

## Author contributions

**Conceptualization:** Jin Hyuck Lee, Jong-Hoon Park, Woo Young Jang.

**Data curation:** Jin Hyuck Lee.

**Formal analysis:** Jin Hyuck Lee.

**Supervision:** Jong-Hoon Park.

**Writing – original draft:** Jin Hyuck Lee, Jong-Hoon Park.

**Writing – review & editing:** Woo Young Jang.

Woo Young Jang orcid: 0000-0003-1775-7715.

## References

[1] Cornwall MW McPoil TG Plantar fasciitis: etiology and treatment. J Orthop Sports Phys Ther 1999;29:756–60. 10612073 10.2519/jospt.1999.29.12.756

[2] Martin RL Davenport TE Reischl SF Heel pain—plantar fasciitis: revision 2014. J Orthop Sports Phys Ther 2014;44:A1–33. 10.2519/jospt.2014.0303 25361863

[3] Buchbinder R Plantar fasciitis. N Engl J Med 2004;350:2159–66. 15152061 10.1056/NEJMcp032745

[4] Riddle DL Pulisic M Pidcoe P Risk factors for Plantar fasciitis: a matched case-control study. J Bone Joint Surg Am 2003;85-a:872–7. 10.2106/00004623-200305000-00015 12728038

[5] Patel A DiGiovanni B Association between plantar fasciitis and isolated contracture of the gastrocnemius. Foot Ankle Int 2011;32:5–8. 21288428 10.3113/FAI.2011.0005

[6] van Leeuwen KD Rogers J Winzenberg T Higher body mass index is associated with plantar fasciopathy/’plantar fasciitis’: systematic review and meta-analysis of various clinical and imaging risk factors. Br J Sports Med 2016;50:972–81. 26644427 10.1136/bjsports-2015-094695

[7] Pollack Y Shashua A Kalichman L Manual therapy for plantar heel pain. Foot (Edinb) 2018;34:11–6. 29175715 10.1016/j.foot.2017.08.001

[8] Tong JW Kong PW Association between foot type and lower extremity injuries: systematic literature review with meta-analysis. J Orthop Sports Phys Ther 2013;43:700–14. 23756327 10.2519/jospt.2013.4225

[9] Sahin N Ozturk A Atici T Foot mobility and plantar fascia elasticity in patients with plantar fasciitis. Acta Orthop Traumatol Turc 2010;44:385–91. 21343689 10.3944/AOTT.2010.2348

[10] Steinberg N Dar G Dunlop M The relationship of hip muscle performance to leg, ankle and foot injuries: a systematic review. Phys Sportsmed 2017;45:49–63. 28067582 10.1080/00913847.2017.1280370

[11] Petersen W Ellermann A Gosele-Koppenburg A Patellofemoral pain syndrome. Knee Surg Sports Traumatol Arthrosc 2014;22:2264–74. 24221245 10.1007/s00167-013-2759-6 PMC4169618

[12] Kibler WB Goldberg C Chandler TJ Functional biomechanical deficits in running athletes with plantar fasciitis. Am J Sports Med 1991;19:66–71. 1672577 10.1177/036354659101900111

[13] Chang CH Miller F Schuyler J Dynamic pedobarograph in evaluation of varus and valgus foot deformities. J Pediatr Orthop 2002;22:813–8. 12409913

[14] Barouk P Barouk LS Clinical diagnosis of gastrocnemius tightness. Foot Ankle Clin 2014;19:659–67. 25456715 10.1016/j.fcl.2014.08.004

[15] Lee KM Chung CY Park MS Reliability and validity of radiographic measurements in hindfoot varus and valgus. J Bone Joint Surg Am 2010;92:2319–27. 20926727 10.2106/JBJS.I.01150

[16] Lee DY Seo SG Kim EJ Correlation between static radiographic measurements and intersegmental angular measurements during gait using a multisegment foot model. Foot Ankle Int 2015;36:1–0. 25404757 10.1177/1071100714559727

[17] De Boer AS Meuffels DE Van der Vlies CH Validation of the American Orthopaedic Foot and Ankle Society Ankle-Hindfoot Scale Dutch language version in patients with hindfoot fractures. BMJ Open 2017;7:e018314. 10.1136/bmjopen-2017-018314 PMC5695419 29138208

[18] McClinton SM Cleland JA Flynn TW Predictors of response to physical therapy intervention for plantar heel pain. Foot Ankle Int 2015;36:408–16. 25367253 10.1177/1071100714558508

[19] Cleland JA Abbott JH Kidd MO Manual physical therapy and exercise versus electrophysical agents and exercise in the management of plantar heel pain: a multicenter randomized clinical trial. J Orthop Sports Phys Ther 2009;39:573–85. 19687575 10.2519/jospt.2009.3036

[20] Buldt AK Allan JJ Landorf KB The relationship between foot posture and plantar pressure during walking in adults: a systematic review. Gait Posture 2018;62:56–67. 29524798 10.1016/j.gaitpost.2018.02.026

[21] Bolgla LA Malone TR Plantar fasciitis and the windlass mechanism: a biomechanical link to clinical practice. J Athl Train 2004;39:77. 16558682 PMC385265

[22] Kirby KA Longitudinal arch load-sharing system of the foot. Rev Españ Podolog 2017;28:e18–26.

[23] Allen RH Gross MT Toe flexors strength and passive extension range of motion of the first metatarsophalangeal joint in individuals with plantar fasciitis. J Orthop Sports Phys Ther 2003;33:468–78. 12968860 10.2519/jospt.2003.33.8.468

[24] Lewis CL Ferris DP Walking with increased ankle pushoff decreases hip muscle moments. J Biomech 2008;41:2082–9. 18606419 10.1016/j.jbiomech.2008.05.013 PMC2562040

[25] Mueller MJ Sinacore DR Hoogstrate S Hip and ankle walking strategies: effect on peak plantar pressures and implications for neuropathic ulceration. Arch Phys Med Rehabil 1994;75:1196–200. 7979928 10.1016/0003-9993(94)90004-3

[26] Lee S-P Powers CM Individuals with diminished hip abductor muscle strength exhibit altered ankle biomechanics and neuromuscular activation during unipedal balance tasks. Gait Posture 2014;39:933–8. 24373699 10.1016/j.gaitpost.2013.12.004

